# Remission, relapse, and risk of major cardiovascular events after metabolic surgery in persons with hypertension: A Swedish nationwide registry-based cohort study

**DOI:** 10.1371/journal.pmed.1003817

**Published:** 2021-11-01

**Authors:** Erik Stenberg, Richard Marsk, Magnus Sundbom, Johan Ottosson, Tomas Jernberg, Ingmar Näslund, Erik Näslund

**Affiliations:** 1 Department of Surgery, Faculty of Medicine and Health, Örebro University, Örebro, Sweden; 2 Division of Surgery, Department of Clinical Sciences, Danderyd Hospital, Karolinska Institutet, Stockholm, Sweden; 3 Department of Surgical Sciences, Uppsala University, Uppsala, Sweden; 4 Division of Cardiovascular Medicine, Department of Clinical Sciences, Danderyd Hospital, Karolinska Institutet, Stockholm, Sweden; Harvard Medical School, UNITED STATES

## Abstract

**Background:**

Several studies have shown that metabolic surgery is associated with remission of diabetes and hypertension. In terms of diabetes, factors such as duration, insulin use, weight loss, and age have been shown to contribute to the likelihood of remission. Such factors have not been determined for hypertension. The aim of this study was to evaluate factors associated with the remission and relapse of hypertension after metabolic surgery, as well as the risk for major adverse cardiovascular event (MACE) and mortality in patients with and without remission.

**Methods and findings:**

All adults who underwent metabolic surgery between January 2007 and June 2016 were identified in the nationwide Scandinavian Obesity Surgery Registry (SOReg). Through cross-linkage with the Swedish Prescribed Drug Register, Patient Register, and Statistics Sweden, individual data on prescriptions, inpatient and outpatient diagnoses, and mortality were retrieved. Of the 15,984 patients with pharmacologically treated hypertension, 6,286 (39.3%) were in remission at 2 years. High weight loss and male sex were associated with higher chance of remission, while duration, number of antihypertensive drugs, age, body mass index (BMI), cardiovascular disease, and dyslipidemia were associated with lower chance. After adjustment for age, sex, BMI, comorbidities, and education, the cumulative probabilities of MACEs (2.8% versus 5.7%, adjusted odds ratio (OR) 0.60, 95% confidence interval (CI) 0.47 to 0.77, *p* < 0.001) and all-cause mortality (4.0% versus 8.0%, adjusted OR 0.71, 95% CI 0.57 to 0.88, *p* = 0.002) were lower for patients being in remission at 2 years compared with patients not in remission, despite relapse of hypertension in 2,089 patients (cumulative probability 56.3%) during 10-year follow-up. The main limitations of the study were missing information on nonpharmacological treatment for hypertension and the observational study design.

**Conclusions:**

In this study, we observed an association between high postoperative weight loss and male sex with better chance of remission, while we observed a lower chance of remission depending on disease severity and presence of other metabolic comorbidities. Patients who achieved remission had a halved risk of MACE and death compared with those who did not. The results suggest that in patients with severe obesity and hypertension, metabolic surgery should not be delayed.

## Abstract 2

In a cohort study, Erik Stenberg and colleagues investigate the association between remission, relapse and risk of major cardiovascular events and metabolic surgery in persons with hypertension in Sweden.

## Author summary

**Why was this study done?:**

- Hypertension, particularly in combination with morbid obesity, is a leading cause of mortality and disability worldwide.
- There is a growing body of evidence supporting the reduction of major adverse cardiovascular events (MACEs) and mortality among patients with metabolic comorbidities after bariatric surgery. Less is known of the factors associated with remission and relapse of disease as well as the impact on MACEs and morality from reaching remission.
- The main purpose of this study was to assess which factors that contribute to the remission and relapse of hypertension after metabolic surgery, as well as the risk for MACE and mortality in patients who have achieved remission of hypertension.

**What did the researchers do and find?:**

- In this nationwide observational study, 15,984 patients with hypertension undergoing a primary metabolic procedure were included.
- Almost 40% of patients with hypertension experienced remission of hypertension 2 years after surgery. While 56% relapsed over 10-year follow-up, those who reached remission experienced lower probability for MACEs and all-cause mortality compared with those who did not reach remission.

**What do these findings mean?:**

- The results of this study suggest that metabolic surgery has the highest success rate for patients early in the course of disease and thus suggests that metabolic surgery should not be delayed for patients with severe obesity and hypertension.

## Introduction

The association between metabolic surgery and remission of type 2 diabetes (T2D) is well established by several randomized controlled trials (RCTs) and observational studies, demonstrating that metabolic surgery is superior to medical treatment of T2D [1–4]. The association between metabolic surgery and the remission of hypertension is less well studied. In an RCT with remission of hypertension as primary outcome measure, 51% of the surgically treated patients achieved remission of their hypertension, which was superior to medical treatment [5]. In an observational study with 12-year follow-up by Adams and colleagues [6], the surgically treated group had both higher remission rates and lower incidence rates of hypertension than persons with severe obesity not undergoing surgery. A recent meta-analysis concluded that remission rates of hypertension were higher in patients that underwent Roux-en-Y gastric bypass (RYGB) compared with sleeve gastrectomy (SG) [7].

Duration of diabetes, glycemic control, use of insulin, age, and postoperative weight loss have all been shown to be associated with the chance of diabetes remission [1,3,4,8–12]. In terms of hypertension, this has been less studied. The Swedish Obese Subjects (SOS) study reported that the proportion of patients on antihypertensive medications were lower in the surgical group compared with the nonoperated matched controls. They found a significant linear positive relationship between change in blood pressure and change in body mass index (BMI) at 2 years of follow-up but not at 10 years of follow-up [13].

It has recently been shown that metabolic surgery in patients with severe obesity and pharmacologically treated hypertension was associated with lower risk for major adverse cardiovascular events (MACEs) and all-cause mortality compared with age- and sex-matched controls with hypertension from the general population [14]. The impact of postoperative remission of hypertension was not clarified.

The aims of this study were to assess which factors are associated with remission and relapse of hypertension after metabolic surgery as well as to compare the risk of MACE and mortality in patients with and without subsequent remission of their hypertension.

## Methods

This study was conducted using a register linkage based on the Scandinavian Obesity Surgery Registry (SOReg), linked to nationwide Swedish health registers, using the unique personal identity number assigned to each Swedish resident. All data were pseudonymized prior to access.

### Study population and intervention

The SOReg is a national register for bariatric and metabolic surgery, covering more than 97.5% of all bariatric surgery procedures in Sweden [15]. All adults (≥18 years of age) who underwent a primary RYGB or SG at some point between January 1, 2007 and June 30, 2016 with a registration in the SOReg were identified and considered for inclusion. The study used the same study base as that of a recent study [14], with the difference that the present study included all patients with hypertension who underwent a primary gastric bypass or SG procedure in Sweden as registered in the SOReg without matching to controls from the general population.

A cross-linkage was performed with the nationwide Swedish Prescribed Drug Register, the National Patient Register (NPR), the Cause of Death Register, and individual socioeconomic data from Statistics Sweden. The Swedish Prescribed Drug Register was established in 2005 and includes all dispensed prescription drugs (updated monthly) classified according to the World Health Organization Anatomical Therapeutic Chemical (ATC) classification system. The Drug Register was complete up until December 31, 2018 at time of retrieval. The NPR is a nationwide register to which hospitals are obliged to report all inpatient and outpatient hospital care data [16]. The inpatient component of the register attained national coverage in 1987 and covers nearly 100% of all hospital admissions in public healthcare, while the outpatient component, started in 2001, has successively reached about 96% of outpatient visits in specialized healthcare. Data from the NPR were available until December 31, 2017. The Total Population Register, continually updated by Statistics Sweden, provides data on emigration/immigration, marriage/divorce, and complete coverage of dates of birth/death for each individual in Sweden [17]. The Education Register from Statistics Sweden provided data on educational levels at the time of surgery. Perioperative data, information on postoperative complications, and weight loss were based on data from the SOReg.

### Inclusion and exclusion criteria

**Fig 1:**
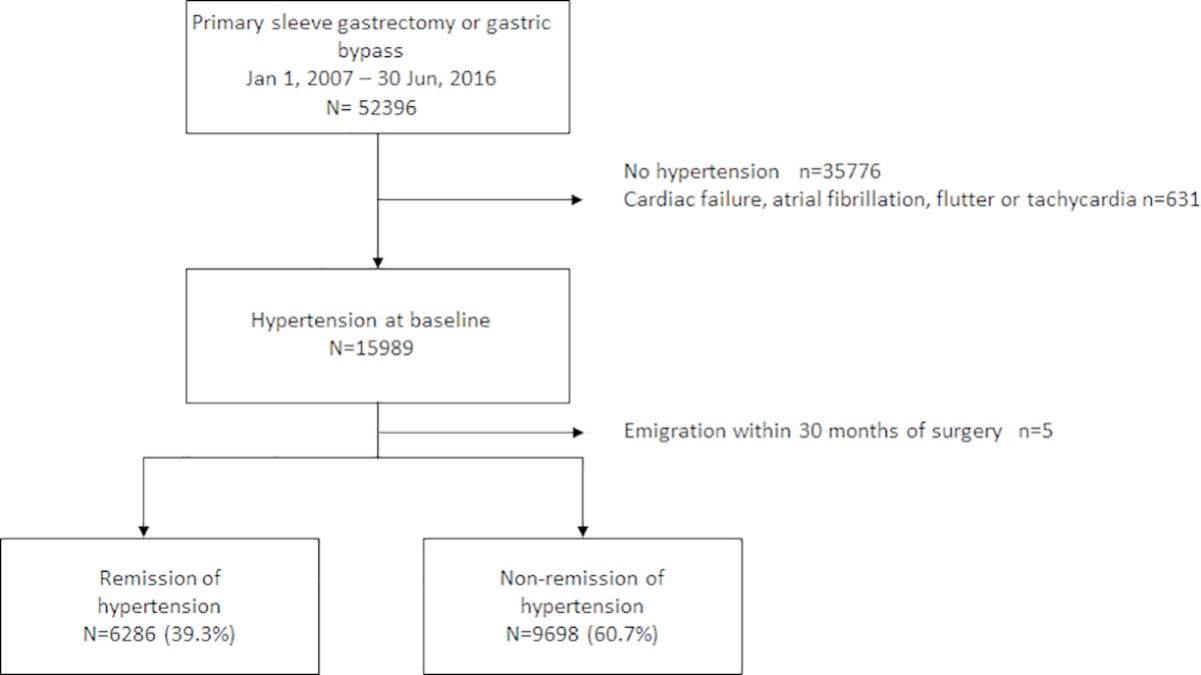
Flowchart describing participant’s identification and inclusion.

There were no mandatory national eligibility criteria for bariatric surgery during the study period, although most regions in Sweden considered BMI ≥35 kg/m^2^ with or without obesity-related comorbidity as eligibility criteria. Hypertension was defined by the use of antihypertensive drugs (ATC codes C02, C03, C07, C08, or C09) within 18 months prior to surgery. As some of these drugs also are used in treating heart failure, atrial fibrillation, and tachycardia, individuals with the following combinations of diagnoses and prescriptions were excluded: heart failure (ICD-10: I50) or cardiomyopathy (ICD-10: I42) and C03C, C07A, C09A, C09B, or C09C; atrial fibrillation, flutter, or other tachycardia (ICD-10: I47 and I48) and C07 or C08D. Patients without hypertension at the time of surgery were also excluded (Fig 1).

### Covariates

Educational level was retrieved from the Education Register at Statistics Sweden. Dispensed prescription drugs were retrieved from the Prescribed Drug Register. Baseline BMI and the presence of sleep apnea, depression, and diabetes were based on data from the SOReg and defined as active treatment (continuous positive airway pressure and pharmacological treatment, respectively) at the time of surgery. The definitions of dyslipidemia and cardiovascular comorbidity were based on data from the Swedish Prescribed Drug Register. Dyslipidemia was defined by the use of lipid modifying drugs (ATC code C10). Diabetes was defined as being prescribed antidiabetic drugs (ATC code A10). Cardiovascular comorbidity was defined as a previous diagnose of heart failure (ICD-10: I50), acute myocardial infarction or angina pectoris (ICD-10: I20 to I22), atrial fibrillation, flutter, or other tachycardia (ICD-10: I47 and I48) not excluded based on the exclusion criteria as listed above. Cerebrovascular disease was defined as a previous cerebrovascular event (ICD-10: I60 to I64).

### Procedures

The surgical technique for the laparoscopic gastric bypass procedure is highly standardized in Sweden with the majority being an antecolic, antegastric RYGB with a small gastric pouch (<25 mL), an alimentary limb of 100 cm, and a biliopancreatic limb of 50 cm [18]. The surgical technique for the laparoscopic SG is less standardized, but routinely performed using a 32–36 Fr bougie, starting the resection no more than 5 cm from the pylorus, ending the resection 1 cm from the angle of His.

### Outcome and follow-up

The main outcome was the remission of pharmacological treatment of hypertension defined as no prescribed antihypertensive drugs for 1 entire year (18 to 30 months after surgery). Patients who died during the first 30 months after surgery were included in the analyses on remission (and considered to not reach remission), but based on this definition, they were excluded from the analyses on mortality, MACE, and relapse since their inclusion would likely overestimate the impact of remission.

Secondary outcomes were all-cause mortality and MACEs, defined as the first occurrence of unstable angina, myocardial infarction, acute cerebrovascular event, fatal cardiovascular event (cause of death ICD-10: I01 to I78, excluding I30), or unattended sudden cardiac death (ICD-10: R96.0, R96.1, R98, and R99). Any adverse event during the first 30 days after surgery were considered to be a postoperative complication. The Clavien–Dindo classification of postoperative complications was introduced in the SOReg on January 1, 2010 [19]. Complications graded as ≥3b (i.e., a complication requiring intervention under general anesthesia, resulting in organ failure or death) were considered to be serious complications. A tertiary outcome was improvement in hypertension estimated as a reduction of at least 30% of the total number of antihypertensive medications [20].

### Statistics

Postoperative weight loss is presented as change in BMI (BMI loss = initial BMI − postoperative BMI), percentage total weight loss (TWL = 100 × weight loss/preoperative weight), and percentage excess BMI loss (EBMIL = 100 × [initial BMI–postoperative BMI]/[initial BMI– 25]). Categorical data are presented as numbers (*n*) and percentage (%), continuous variables as mean +/− standard deviation (SD). Differences in weight loss depending on surgical method was evaluated using the Student *t* test. Univariable logistic regression and multivariable logistic regression (including age, BMI, duration of antihypertensive treatment (more recent or longer than 1 year), and severity of hypertension (number antihypertensive drug prescribed at baseline), age, sex, comorbidities (dyslipidemia, sleep apnea, depression, diabetes, and cardiovascular and cerebrovascular comorbidities), and TWL at 1 year after surgery) were used to assess the chance of reaching remission, reporting odds ratios (ORs) with 95% confidence intervals (CIs) as measures of association. Risk for relapse of hypertension was estimated using univariable and multivariable Cox regression (including the same variables as listed above). Time to relapse and first episodes of MACE and all-cause mortality were estimated and visualized using the Kaplan–Meier method. Comparisons were made by the log-rank test. Risks for MACE and mortality were estimated using univariable and multivariable Cox regression including factors potentially affecting the risk for these outcomes (adjusted for age, sex, BMI, dyslipidemia, depression, sleep apnea, diabetes, cardiovascular and cerebrovascular comorbidities, and education). The regression models were tested for multicollinearity using linear regression with a variance inflation factor >5 considered to indicate an issue with multicollinearity. No multicollinearity issues were detected in either of the multivariable models. Participants were followed from the surgery date until emigration, death, or end of follow-up, whichever came first. The Wilcoxon signed rank test was used to evaluate differences in numbers of antihypertensive medications.

Although an original study plan was decided on by the authors, it was not officially documented beforehand. A post hoc analysis (not within the original study plan) in the form of secondary analyses on remission and relapse were made stratified by sex, while weight loss results were stratified by surgical method. In addition, the numbers of antihypertensive drugs before and after surgery were presented in numbers in S3 Table. A tertiary analysis concerning improvement of hypertension was included to allow comparison to other important recent studies in the field. Finally, the Bonferroni–Holm method was included to adjust for multiple calculations separately in all of the multivariable analyses [21].

IBM SPSS version 25 (IBM, Armonk, New York, United States of America) was used for all statistical analyses.

### Ethics

The study was approved by the regional ethical review board of Stockholm (ref nr 2017/857-32). No written consent was obtained from the study participants. However, in accordance with Swedish legislation, all participants were informed of the research and quality registry and that the data will be used in clinical research, giving the patients the right to deny participation.

This study is reported as per the Strengthening the Reporting of Observational Studies in Epidemiology (STROBE) guideline (S1 Checklist).

## Results

During the study period, 52,396 patients were identified. After exclusion of patients with heart failure, atrial fibrillation and tachycardia, and patients without hypertension at baseline, 15,989 patients were included in the study cohort. After exclusion of 5 patients who emigrated within 30 months of surgery, 15,984 remained in the study (Fig 1).

Follow-up for 30-day complications was 98.7% (*n* = 15,778). The follow-up rate for weight loss was 89.7% (*n* = 14,332) at 1 year and 59.8% (*n* = 9,559) at 2 years after surgery. All patients were followed for mortality and the use of prescribed drugs until the end of the study. Mean follow-up time was 6.5 ± 2.3 years. Baseline characteristics for the study group is presented in Table 1.

**Table 1:** Baseline cha racteristics of the study cohort.

	Missing data	Entire cohort	Remission at 2 years	Nonremission at 2 years	*p*-Value^1^
Total number of patients		15,984	6,286 (39.3%)	9,698 (60.7%)	
Hypertension duration >1 year, *n* (%)	0 (0.0%)	13,268 (83.0%)	4,406 (33.2%)	8,862 (66.8%)	<0.001
Numbers of preoperative drugs, *n* (%)	0 (0.0%)				
1		5,864 (36.7%)	3,624 (61.8%)	2,240 (38.2%)	Reference
2		4,524 (28.3%)	1,650 (36.5%)	2,874 (63.5%)	<0.001
3		3,149 (19.7%)	708 (22.5%)	2,441 (77.5%)	<0.001
4		1,646 (10.3%)	240 (14.6%)	1,406 (85.4%)	<0.001
≥5		801 (5.0%)	64 (8.0%)	737 (92.0%)	<0.001
Age, mean ± SD	0 (0.0%)	48.7 ± 9.0	45.5 ± 9.2	50.8 ± 8.2	<0.001
BMI, mean ± SD	0 (0.0%)	42.2 ± 5.6	42.2 ± 5.7	42.3 ± 5.6	0.505
Sex, *n* (%)	0 (0.0%)				
Female		10,608 (66.4%)	4,338 (40.9%)	6,270 (59.1%)	Reference
Male		5,376 (33.6%)	1,948 (36.2%)	3,428 (63.8%)	<0.001
Comorbid disease, *n* (%)	0 (0.0%)				
Dyslipidemia		5,208 (32.6%)	1,395 (26.8%)	3,813 (73.2%)	<0.001
Depression		2,694 (16.9%)	1,028 (38.2%)	1,666 (61.8%)	0.174
Sleep apnea		2,732 (17.1%)	901 (33.0%)	1,831 (67.0%)	<0.001
Diabetes		4,989 (31.2%)	1,580 (31.7%)	3,409 (68.3%)	<0.001
Cardiovascular comorbidity		970 (6.1%)	125 (12.9%)	845 (87.1%)	<0.001
Cerebrovascular disease		316 (2.0%)	64 (20.3%)	252 (79.7%)	<0.001
Education, *n* (%)	59 (0.4%)				
Primary education		2,896 (18.2%)	1,082 (37.4%)	1,814 (62.6%)	0.022
Secondary education		9,419 (59.1%)	3,743 (39.7%)	5,676 (60.3%)	Reference
Higher education		3,610 (22.7%)	1,435 (39.8%)	2,175 (60.2%)	0.990
Surgical method, *n* (%)	0 (0.0%)				
Gastric bypass		14,821 (92.7%)	5,886 (39.7%)	8,935 (60.3%)	Reference
SG		1,163 (7.3%)	400 (34.4%)	763 (65.6%)	<0.001

^1^ *p*-Value based on unadjusted logistic regression.

BMI, body mass index (in kg/m^2^); *n*, numbers; SD, standard deviation; SG, sleeve gastrectomy.

### Surgery and weight outcome

The majority of the surgical procedures were laparoscopic 15,365 (96.1%), with conversion to open surgery in 172 operations (1.1%), while the remaining 447 (2.8%) procedures were primarily performed using an open technique. A postoperative complication of any type occurred for 1,531 patients (9.7%) within 30 days after surgery, and of the 13,403 patients operated after January 1, 2010 with a follow-up at day 30, 472 (3.5%) experienced a serious postoperative complication. Mean BMI loss 1 year after surgery was 12.6 ± 4.0 kg/m^2^, with TWL 29.6± 7.8% and EBMIL 76.2 ± 23.0%. Mean BMI loss 2 years after surgery was 12.7 ± 4.6 kg/m^2^, with TWL 29.9± 9.1% and EBMIL 76.7 ± 24.8%. When stratified by surgical method, the mean BMI loss 1 year after surgery was 12.8 ± 3.9 kg/m^2^ versus 9.9 ± 4.2 kg/m^2^, with TWL 30.0 ± 7.6% versus 24.2 ± 8.7%, and EBMIL 76.8 ± 22.5% versus 67.5 ± 27.1% for gastric bypass and SG, respectively (*p* < 0.001). Mean BMI loss at 2 years after surgery was 12.9 ± 4.5 kg/m^2^ versus 9.1 ± 4.7 kg/m^2^, TWL 30.3 ± 8.9% versus 22.6 ± 10.1%, and EBMIL 77.3 ± 24.3% versus 63.3 ± 30.7%, respectively (*p* < 0.001).

### Remission of pharmacological treatment of hypertension

At 2 years after surgery, 6,286 patients (39.3%) had been taken off their pharmacological treatment for hypertension. The duration of disease, number of antihypertensive drugs, age, BMI, dyslipidemia, and cardiovascular disease were all associated with a lower remission rate, while higher postoperative weight loss and male sex were associated with a higher chance of reaching remission (Table 2, S1 Table).

**Table 2:** Chance of reaching hypertension remission 2 years after surgery.

	Unadjusted OR	Adjusted OR	Adjusted *p*-value^1^
Hypertension duration >1 year	0.22 (0.20 to 0.24)	0.41 (0.37 to 0.46)	<0.001*
Numbers of preoperative drugs			
1	Reference	Reference	Reference
2	0.35 (0.33 to 0.38)	0.45 (0.41 to 0.50)	<0.001*
3	0.18 (0.16 to 0.20)	0.26 (0.23 to 0.29)	<0.001*
4	0.11 (0.09 to 0.12)	0.16 (0.13 to 0.18)	<0.001*
≥5	0.05 (0.04 to 0.07)	0.09 (0.07 to 0.12)	<0.001*
%TWL, 1 year after surgery	1.05 (1.04 to 1.05)	1.04 (1.03 to 1.04)	<0.001*
Age	0.93 (0.93 to 0.94)	0.96 (0.96 to 0.97)	<0.001*
BMI	1.00 (0.99 to 1.00)	0.98 (0.97 to 0.98)	<0.001*
Sex			
Female	Reference	Reference	Reference
Male	0.82 (0.77 to 0.88)	1.20 (1.10 to 1.32)	<0.001*
Comorbid disease			
Dyslipidemia	0.44 (0.41 to 0.47)	0.87 (0.78 to 0.95)	0.004*
Depression	0.94 (0.87 to 1.03)	0.93 (0.84 to 1.03)	0.186
Sleep apnea	0.72 (0.66 to 0.78)	1.00 (0.89 to 1.11)	0.932
Diabetes	0.59 (0.55 to 0.64)	1.02 (0.93 to 1.13)	0.625
Cardiovascular comorbidity	0.21 (0.18 to 0.26)	0.45 (0.36 to 0.56)	<0.001*
Cerebrovascular disease	0.39 (0.29 to 0.51)	1.03 (0.74 to 1.45)	0.850
Education			
Primary education	0.90 (0.83 to 0.99)	1.05 (0.95 to 1.17)	0.323
Secondary education	Reference	Reference	Reference
Higher education	1.00 (0.93 to 1.08)	1.02 (0.93 to 1.12)	0.633
Surgical method			
Gastric bypass	Reference	Reference	Reference
SG	0.80 (0.70 to 0.90)	0.87 (0.74 to 1.01)	0.075

^1^ Multivariable, logistic regression model, including all variables in the table.

* Significant value after correction with the Bonferroni–Holm method.

%TWL, percentage total weight loss; BMI, body mass index; CI, confidence interval; OR, odds ratio (presented with 95% CI); *n*, numbers; SG, sleeve gastrectomy.

The number of antihypertensive medications was reduced from a median of 2 (IQR 1 to 3) before surgery to a median of 1 (IQR 0 to 2) after surgery (*p* < 0.001). A reduction of at least 30% of the numbers of antihypertensive drugs was seen in 10,290 patients (64.9%) at 2 years after surgery (S3 Table, S1 Fig).

### Relapse of hypertension

**Fig 2:**
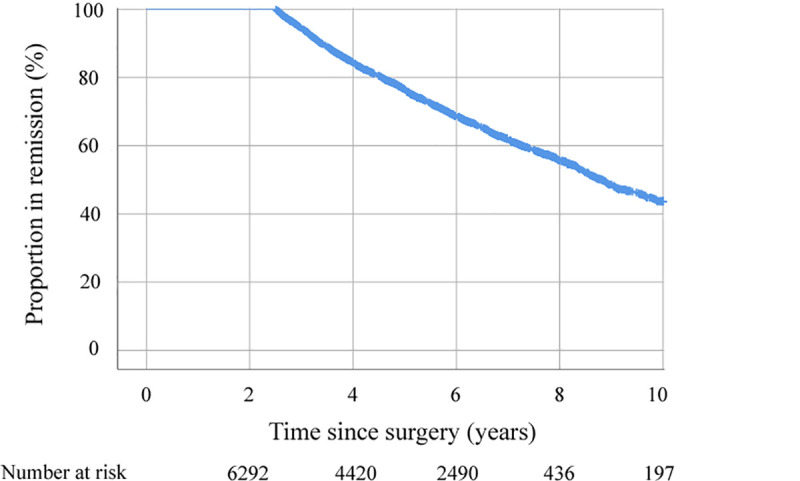
Relapse-free survival for patients experiencing remission of hypertension at 2 years after metabolic surgery (unadjusted Kaplan–Meier curve).

Of the 6,286 patients who initially reached remission, relapse occurred in 2,089 patients within 10 years (cumulative probability 56.3 ± 2.4%) (Fig 2). The duration of disease, number of antihypertensive drugs, age, and SG were all associated with a higher risk for relapse, while higher postoperative weight loss was associated with a lower risk for relapse (Table 3, S2 Table).

**Table 3:** Factors associated with risk for relapse of hypertension.

	Unadjusted HR	Adjusted HR	Adjusted *p*-value^1^
Hypertension duration >1 year	1.69 (1.53 to 1.88)	1.46 (1.30 to 1.63)	<0.001*
Numbers of preoperative drugs			
1	Reference	Reference	Reference
2	1.58 (1.43 to 1.75)	1.39 (1.25 to 1.55)	<0.001*
3	2.20 (1.95 to 2.48)	1.83 (1.59 to 2.09)	<0.001*
4	2.53 (2.11 to 3.04)	2.33 (1.92 to 2.84)	<0.001*
≥5	3.61 (2.63 to 4.96)	2.99 (2.11 to 4.25)	<0.001*
%TWL 1 year after surgery	0.98 (0.98 to 0.99)	0.98 (0.98 to 0.99)	<0.001*
Age	1.02 (1.02 to 1.03)	1.01 (1.01 to 1.02)	<0.001*
BMI	1.00 (0.99 to 1.00)	1.01 (1.00 to 1.02)	0.050
Sex			
Female	Reference	Reference	Reference
Male	1.14 (1.04 to 1.24)	1.00 (0.90 to 1.11)	0.992
Comorbid disease			
Dyslipidemia	1.23 (1.12 to 1.36)	0.96 (0.85 to 1.08)	0.475
Depression	1.08 (0.96 to 1.21)	1.06 (0.93 to 1.20)	0.364
Sleep apnea	1.17 (1.04 to 1.31)	1.04 (0.91 to 1.18)	0.573
T2D	1.17 (1.06 to 1.28)	1.05 (0.94 to 1.18)	0.379
Cardiovascular comorbidity	1.55 (1.19 to 2.03)	1.07 (0.78 to 1.48)	0.663
Cerebrovascular disease	1.53 (1.06 to 2.19)	1.24 (0.81 to 1.90)	0.328
Education			
Primary education	1.12 (1.00 to 1.25)	1.08 (0.95 to 1.22)	0.245
Secondary education	Reference	Reference	Reference
Higher education	0.94 (0.89 to 1.11)	0.95 (0.85 to 1.07)	0.418
Surgical method			
Gastric bypass	Reference	Reference	Reference
SG	1.47 (1.17 to 1.85)	1.48 (1.16 to 1.90)	0.002*

^1^ Multivariable Cox regression model, including all variables in the table.

* Significant value after correction with the Bonferroni–Holm method.

%TWL, percentage total weight loss; BMI, body mass index; CI, confidence interval; HR, hazard ratio (presented with 95% CI); *n*, numbers; SG, sleeve gastrectomy; T2D, type 2 diabetes.

### MACEs

**Fig 3:**
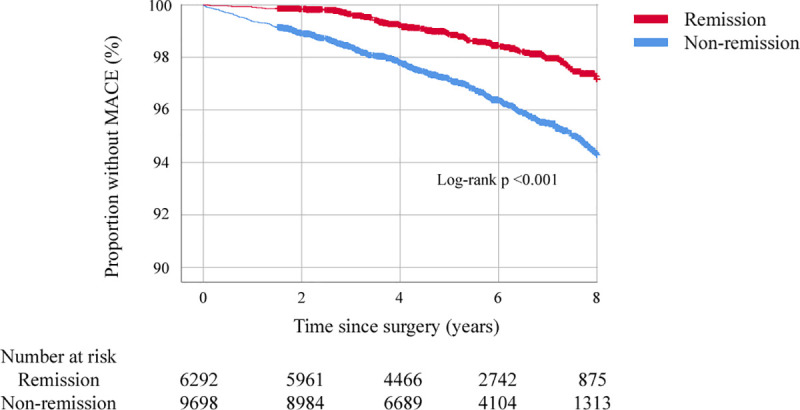
MACE-free survival in patients experiencing remission of hypertension at 2 years and those not experiencing remission (unadjusted Kaplan–Meier curve). MACE, major adverse cardiovascular event.

At 8 years after surgery, MACEs had occurred for 90 patients who initially reached remission (cumulative probability 2.8 ± 0.6%), compared with 366 patients who did not reach remission (cumulative probability 5.7 ± 0.8%): unadjusted hazard ratio (HR) 0.42 (95% CI 0.33 to 0.53, *p* < 0.001) and adjusted HR 0.60 (95% CI 0.47 to 0.77, adjusted *p* < 0.001) (Fig 3, S4 Table).

### Mortality

**Fig 4:**
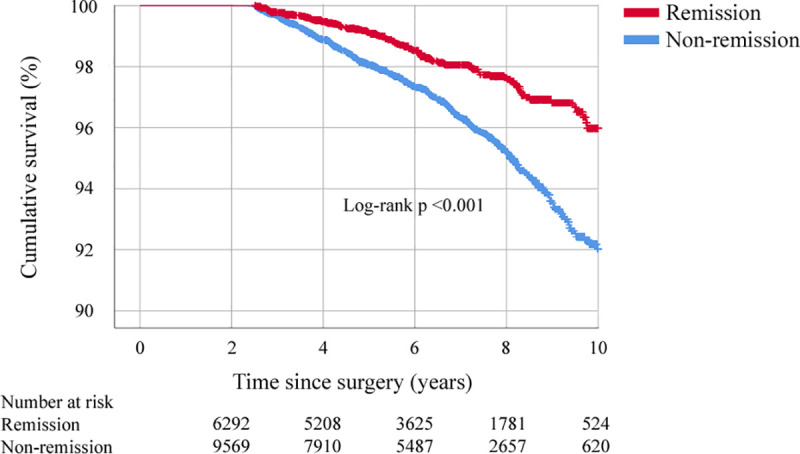
Overall survival in patients experiencing remission of hypertension at 2 years and those not experiencing remission (unadjusted Kaplan–Meier curve).

At 10 years after surgery, 115 patients who initially reached remission had died (cumulative probability 4.0 ± 1.0%), compared with 345 patients who did not reach remission (cumulative probability 8.0 ± 1.2%): unadjusted HR 0.51 (95% CI 0.41 to 0.62, *p* < 0.001) and adjusted HR 0.71 (95% CI 0.57 to 0.88, adjusted *p* = 0.002) (Fig 4, S5 Table).

## Discussion

This study has demonstrated that disease severity, duration, degree of weight loss, age, sex, and metabolic comorbidities are all factors associated with the chance of remission of hypertension after metabolic surgery. The risk of relapse is associated with severity of disease, duration, degree of weight loss, age as well as surgical procedure. Patients who achieve remission of their hypertension 2 years after metabolic surgery have a lower risk of MACE and mortality than those who do not.

There is a high degree of similarity between factors associated with the remission of T2D and the factors found to be associated with the remission of hypertension in this study [4]. Both are chronically progressive diseases with obesity as a shared risk factor [22]. Similar to the effects of metabolic surgery on T2D [23], the effect on blood pressure is seen as early as in the first week after surgery [24], thus suggesting that the cardiovascular and metabolic improvements may at least partially be mediated by similar mechanisms. Although the degree of weight loss was found to be associated with the chance of remission of hypertension in this study, as earlier shown for T2D [4], the effect on both hypertension and T2D occurs before any significant weight loss has occurred. This, and the recent observation that the risk of MACE decreases after approximately 10% of weight loss in bariatric surgical patients with T2D (of which 82% had hypertension) compared with approximately 20% in a matched nonsurgical group [25], suggests that other factors than just weight loss might be in play. Voluntary, nonsurgical weight loss has previously been associated with increased diuresis and negative potassium and sodium balance during the initial weight loss phase [26]. An increased diuresis after longer follow-up time has also been described after gastric bypass, with a linear association between daily urinary output and effects on blood pressure [13].

We found a 2-year remission rate of hypertension of 39%. This is lower than the 63% resolution or improvement of hypertension reported by Vest and colleagues after a mean follow-up of 5 years in a systematic review [27], but similar to the remission rates reported in the Gastric Bypass to Treat Obese Patients With Steady Hypertension (GATEWAY) trial [20] as well as those reported in a recent Norwegian cohort study [28]. Although it is possible that some patients in our study were maintained on the prescribed medication despite having achieved remission, even patients not reaching remission may well be able to reduce the number of drugs needed to reach sufficient blood pressure levels [14,20]. Using a similar definition of improvement to that of the GATEWAY trial, 65% of the patients in our study were able to reduce their number of antihypertensive medications by at least 30%. This rate compares well to that reported in previous studies [20,27].

The true effect of metabolic surgery on hypertension has been questioned [29], and high rates of relapse have been reported. We found that 56% of those who went into initial remission of hypertension eventually relapsed at 10 years after surgery. Again, the duration and severity of disease were the main factors associated with the risk for relapse. In contrast to remission, the SG was associated with higher risk of relapse even after adjustment for other potential explanatory factors such as postoperative weight loss. Although this finding should be viewed with some caution with respect to the nonrandomized design, it supports the growing body of evidence supporting a better metabolic effect of the gastric bypass procedure that goes beyond strict weight loss effects [30,31]. In the SOS study, the use of antihypertensive medications was reduced from 39% to 27% at 2-year follow-up after RYGB, while no difference was seen at 10 years, suggesting a significant relapse of hypertension. Furthermore, the linear association between the reduction in blood pressure and weight loss at 2 years was not evident at 10-year follow-up. The number of patients who underwent RYGB were, however, few in the SOS study, and only 68% were followed for 10 years [13]. In the present study, the follow-up rate for the pharmacological treatment of hypertension was 100%.

Although the risk for relapse of hypertension is high, patients who went into initial remission also experienced significant reduction in the risk for MACE as well as all-cause mortality. Several recent reports have highlighted a reduced risk of MACE and mortality in patients with T2D or hypertension that undergo metabolic surgery. Aminian and colleagues [31] recently reported a significant difference in favor of metabolic surgery for an extended MACE outcome (adjusted HR 0.61; 95% CI [0.55 to 0.69]) in patients with obesity and T2D. Stenberg and colleagues [14] reported a reduction in risk of MACE after metabolic surgery (adjusted HR 0.73, 95% CI 0.64 to 0.84, *p* < 0.001) in 11,863 patients with obesity and pharmacologically treated hypertension compared with 26,199 matched individuals with hypertension from the general population, after adjustment for duration of hypertension, comorbidities, and education. In light of this, our finding that the risk for MACE and mortality was lower in those who achieved remission of hypertension is not surprising, but it highlights the importance of prioritizing patients having much to gain from metabolic surgery. Further studies focused on optimizing the results for patients who do not reach remission is also needed.

Despite the strengths of the large, nationwide study population and the use of high-quality data from several sources, this study has several limitations. First, this is an observational study. Thus, we cannot exclude that residual confounding factors exist. We, therefore, need to be cautious regarding causality. The study group was limited to patients receiving pharmacological treatment for hypertension, thus excluding patients with undiagnosed hypertension before surgery as well as patients with low adherence to treatment. The results of this study may not be applicable to these specific situations, which deserves the focus of future studies. Furthermore, data on the remission of hypertension after metabolic surgery were based on the discontinuation of pharmacological treatment that may result in an overestimation as well as underestimation of the true remission rates. We, however, believe that underestimation is more likely given the inertia of the healthcare system when it comes to discontinuation of chronic medication. As with all registry studies, there may exist coding errors. On the other hand, this study was based on large nationwide registers with known high validity and degree of completeness.

## Conclusions

This study has demonstrated that metabolic surgery is associated with a significant degree of remission of hypertension with a high postoperative weight loss and male sex being associated with better chance of remission, while age, disease severity, and presence of other metabolic comorbidities being associated with lower chance. Although more than half of the patients relapse within 10 years after surgery, the risks for MACE and mortality are markedly reduced compared with those who do not achieve remission at 2 years. Taken together, this suggests that in patients with severe obesity and hypertension, metabolic surgery should not be delayed.

## Supporting information

S1 Checklist STROBE Checklist. STROBE, Strengthening the Reporting of Observational Studies in Epidemiology.
(DOCX)

S1 Table Multivariable logistic regression model for the chance of reaching hypertension remission at 2 years after surgery stratified by sex. (DOCX)

S2 Table Multivariable Cox regression model for factors associated with risk for relapse of hypertension stratified by sex. (DOCX)

S3 Table Numbers of antihypertensive drugs before and 2 years after surgery. (DOCX)

S4 Table Multivariable Cox regression model for the occurrence of MACE up to 8 years after surgery. MACE, major adverse cardiovascular event.
(DOCX)

S5 Table Multivariable Cox regression model for the occurrence of mortality up to 10 years after surgery. (DOCX)

S1 Fig Stacked histogram of numbers of antihypertensive drugs before and 2 years after surgery. (TIF)

## Abbreviations

ATC: Anatomical Therapeutic Chemical
BMI: body mass index
CI: confidence interval
EBMIL: excess BMI loss
GATEWAY: Gastric Bypass to Treat Obese Patients With Steady Hypertension
HR: hazard ratio
MACE: major adverse cardiovascular event
NPR: National Patient Register
OR: odds ratio
RCT: randomized controlled trial
RYGB: Roux-en-Y gastric bypass
SD: standard deviation
SG: sleeve gastrectomy
SOReg: Scandinavian Obesity Surgery Registry
SOS: Swedish Obese Subjects
STROBE: Strengthening the Reporting of Observational Studies in Epidemiology
T2D: type 2 diabetes
TWL: total weight loss

## References

[1] Schauer PR, Bhatt DL, Kirwan JP, Wolski K, Aminian A, Brethauer SA, et al. Bariatric Surgery versus Intensive Medical Therapy for Diabetes—5-Year Outcomes. N Engl J Med. 2017;376(7):641–51. doi: 10.1056/NEJMoa1600869 ; PubMed Central PMCID: PMC5451258. 28199805 PMC5451258

[2] Mingrone G, Panunzi S, De Gaetano A, Guidone C, Iaconelli A, Nanni G, et al. Bariatric-metabolic surgery versus conventional medical treatment in obese patients with type 2 diabetes: 5 year follow-up of an open-label, single-centre, randomised controlled trial. Lancet. 2015;386(9997):964–73. Epub 2015/09/16. doi: 10.1016/S0140-6736(15)00075-6 . 26369473

[3] Backman O, Bruze G, Naslund I, Ottosson J, Marsk R, Neovius M, et al. Gastric Bypass Surgery Reduces De Novo Cases of Type 2 Diabetes to Population Levels: A Nationwide Cohort Study From Sweden. Ann Surg. 2019;269(5):895–902. Epub 2018/08/14. doi: 10.1097/SLA.0000000000002983 . 30102631 PMC7306254

[4] Jans A, Naslund I, Ottosson J, Szabo E, Naslund E, Stenberg E. Duration of type 2 diabetes and remission rates after bariatric surgery in Sweden 2007–2015: A registry-based cohort study. PLoS Med. 2019;16(11):e1002985. Epub 2019/11/21. doi: 10.1371/journal.pmed.1002985 . 31747392 PMC6867594

[5] Schiavon CA, Bersch-Ferreira AC, Santucci EV, Oliveira JD, Torreglosa CR, Bueno PT, et al. Effects of Bariatric Surgery in Obese Patients With Hypertension: The GATEWAY Randomized Trial (Gastric Bypass to Treat Obese Patients With Steady Hypertension). Circulation. 2018;137(11):1132–42. Epub 2017/11/15. doi: 10.1161/CIRCULATIONAHA.117.032130 ; PubMed Central PMCID: PMC5865494. 29133606 PMC5865494

[6] Adams TD, Davidson LE, Litwin SE, Kim J, Kolotkin RL, Nanjee MN, et al. Weight and Metabolic Outcomes 12 Years after Gastric Bypass. N Engl J Med. 2017;377(12):1143–55. Epub 2017/09/21. doi: 10.1056/NEJMoa1700459 ; PubMed Central PMCID: PMC5737957. 28930514 PMC5737957

[7] Climent E, Goday A, Pedro-Botet J, Solà I, Oliveras A, Ramón JM, et al. Laparoscopic Roux-en-Y gastric bypass versus laparoscopic sleeve gastrectomy for 5-year hypertension remission in obese patients: a systematic review and meta-analysis. J Hypertens. 2020;38(2):185–95. Epub 2019/10/22. doi: 10.1097/HJH.0000000000002255 . 31633582

[8] Arterburn DE, Bogart A, Sherwood NE, Sidney S, Coleman KJ, Haneuse S, et al. A multisite study of long-term remission and relapse of type 2 diabetes mellitus following gastric bypass. Obes Surg. 2013;23(1):93–102. doi: 10.1007/s11695-012-0802-1 ; PubMed Central PMCID: PMC4641311. 23161525 PMC4641311

[9] Panunzi S, De Gaetano A, Carnicelli A, Mingrone G. Predictors of remission of diabetes mellitus in severely obese individuals undergoing bariatric surgery: do BMI or procedure choice matter? A meta-analysis. Ann Surg. 2015;261(3):459–67. doi: 10.1097/SLA.0000000000000863 . 25361217

[10] Aung L, Lee WJ, Chen SC, Ser KH, Wu CC, Chong K, et al. Bariatric Surgery for Patients With Early-Onset vs Late-Onset Type 2 Diabetes. JAMA Surg. 2016;151(9):798–805. doi: 10.1001/jamasurg.2016.1130 . 27248572

[11] Blackstone R, Bunt JC, Cortes MC, Sugerman HJ. Type 2 diabetes after gastric bypass: remission in five models using HbA1c, fasting blood glucose, and medication status. Surg Obes Relat Dis. 2012;8(5):548–55. Epub 2012/06/23. doi: 10.1016/j.soard.2012.05.005 . 22721581

[12] Brethauer SA, Aminian A, Romero-Talamas H, Batayyah E, Mackey J, Kennedy L, et al. Can diabetes be surgically cured? Long-term metabolic effects of bariatric surgery in obese patients with type 2 diabetes mellitus. Ann Surg. 2013;258(4):628–36; discussion 36–7. Epub 2013/09/11. doi: 10.1097/SLA.0b013e3182a5034b ; PubMed Central PMCID: PMC4110959. 24018646 PMC4110959

[13] Hallersund P, Sjostrom L, Olbers T, Lonroth H, Jacobson P, Wallenius V, et al. Gastric bypass surgery is followed by lowered blood pressure and increased diuresis—long term results from the Swedish Obese Subjects (SOS) study. PLoS ONE. 2012;7(11):e49696. doi: 10.1371/journal.pone.0049696 ; PubMed Central PMCID: PMC3510228. 23209592 PMC3510228

[14] Stenberg E, Cao Y, Marsk R, Sundbom M, Jernberg T, Näslund E. Association between metabolic surgery and cardiovascular outcome in patients with hypertension: A nationwide matched cohort study. PLoS Med. 2020;17(9):e1003307. Epub 2020/09/16. doi: 10.1371/journal.pmed.1003307 ; PubMed Central PMCID: PMC7491727. 32931494 PMC7491727

[15] Sundbom M, Näslund I, Näslund E, Ottosson J. High acquisition rate and internal validity in the Scandinavian Obesity Surgery Registry. Surg Obes Relat Dis. 2020. Epub 2020/11/28. doi: 10.1016/j.soard.2020.10.017 . 33243667

[16] Ludvigsson JF, Andersson E, Ekbom A, Feychting M, Kim JL, Reuterwall C, et al. External review and validation of the Swedish national inpatient register. BMC Public Health. 2011;11:450. doi: 10.1186/1471-2458-11-450 ; PubMed Central PMCID: PMC3142234. 21658213 PMC3142234

[17] Ludvigsson JF, Almqvist C, Bonamy AK, Ljung R, Michaëlsson K, Neovius M, et al. Registers of the Swedish total population and their use in medical research. Eur J Epidemiol. 2016;31(2):125–36. Epub 2016/01/16. doi: 10.1007/s10654-016-0117-y . 26769609

[18] Olbers T, Lonroth H, Fagevik-Olsen M, Lundell L. Laparoscopic gastric bypass: development of technique, respiratory function, and long-term outcome. Obes Surg. 2003;13(3):364–70. doi: 10.1381/096089203765887679 . 12841895

[19] Clavien PA, Barkun J, de Oliveira ML, Vauthey JN, Dindo D, Schulick RD, et al. The Clavien-Dindo classification of surgical complications: five-year experience. Ann Surg. 2009;250(2):187–96. doi: 10.1097/SLA.0b013e3181b13ca2 . 19638912

[20] Schiavon CA, Bhatt DL, Ikeoka D, Santucci EV, Santos RN, Damiani LP, et al. Three-Year Outcomes of Bariatric Surgery in Patients With Obesity and Hypertension: A Randomized Clinical Trial. Ann Intern Med 2020;173(9):685–93. Epub 2020/08/18. doi: 10.7326/M19-3781 . 32805133

[21] Holm S. A Simple Sequentially Rejective Multiple Test Procedure. Scand J Stat. 1979;6(2):65–70.

[22] Manson JE, Colditz GA, Stampfer MJ, Willett WC, Rosner B, Monson RR, et al. A prospective study of obesity and risk of coronary heart disease in women. N Engl J Med. 1990;322(13):882–9. Epub 1990/03/29. doi: 10.1056/NEJM199003293221303 . 2314422

[23] Falken Y, Hellstrom PM, Holst JJ, Naslund E. Changes in glucose homeostasis after Roux-en-Y gastric bypass surgery for obesity at day three, two months, and one year after surgery: role of gut peptides. J Clin Endocrinol Metab. 2011;96(7):2227–35. doi: 10.1210/jc.2010-2876 . 21543426

[24] Ahmed AR, Rickards G, Coniglio D, Xia Y, Johnson J, Boss T, et al. Laparoscopic Roux-en-Y gastric bypass and its early effect on blood pressure. Obes Surg. 2009;19(7):845–9. Epub 2008/09/02. doi: 10.1007/s11695-008-9671-z . 18758869

[25] Aminian A, Zajichek A, Tu C, Wolski KE, Brethauer SA, Schauer PR, et al. How Much Weight Loss is Required for Cardiovascular Benefits? Insights From a Metabolic Surgery Matched-cohort Study. Ann Surg. 2020;272(4):639–45. Epub 2020/09/16. doi: 10.1097/SLA.0000000000004369 . 32932320

[26] Heymsfield SB, Thomas D, Nguyen AM, Peng JZ, Martin C, Shen W, et al. Voluntary weight loss: systematic review of early phase body composition changes. Obes Rev. 2011;12(5):e348–61. Epub 2010/06/08. doi: 10.1111/j.1467-789X.2010.00767.x . 20524998

[27] Vest AR, Heneghan HM, Agarwal S, Schauer PR, Young JB. Bariatric surgery and cardiovascular outcomes: a systematic review. Heart. 2012;98(24):1763–77. doi: 10.1136/heartjnl-2012-301778 . 23077152

[28] Jakobsen GS, Smastuen MC, Sandbu R, Nordstrand N, Hofso D, Lindberg M, et al. Association of Bariatric Surgery vs Medical Obesity Treatment With Long-term Medical Complications and Obesity-Related Comorbidities. JAMA. 2018;319(3):291–301. Epub 2018/01/18. doi: 10.1001/jama.2017.21055 ; PubMed Central PMCID: PMC5833560. 29340680 PMC5833560

[29] Livingston EH. Reimagining Obesity in 2018: A JAMA Theme Issue on Obesity. JAMA. 2018;319(3):238–40. Epub 2018/01/18. doi: 10.1001/jama.2017.21779 . 29340660

[30] Lee Y, Doumouras AG, Yu J, Aditya I, Gmora S, Anvari M, et al. Laparoscopic Sleeve Gastrectomy Versus Laparoscopic Roux-en-Y Gastric Bypass: A Systematic Review and Meta-analysis of Weight Loss, Comorbidities, and Biochemical Outcomes From Randomized Controlled Trials. Ann Surg. 2021;273(1):66–74. Epub 2019/11/07. doi: 10.1097/SLA.0000000000003671 . 31693504

[31] Aminian A, Zajichek A, Arterburn DE, Wolski KE, Brethauer SA, Schauer PR, et al. Association of Metabolic Surgery With Major Adverse Cardiovascular Outcomes in Patients With Type 2 Diabetes and Obesity. JAMA. 2019;322(13):1271–82. Epub 2019/09/03. doi: 10.1001/jama.2019.14231 ; PubMed Central PMCID: PMC6724187. 31475297 PMC6724187

