# Supplementary material for: Remission, relapse, and risk of major cardiovascular events after metabolic surgery in persons with hypertension: A Swedish nationwide registry-based cohort study
Source: PLoS Med. 2021 Nov 1;18(11):e1003817. doi: 10.1371/journal.pmed.1003817 (PMC8559928; doi:10.1371/journal.pmed.1003817)
Supplement: S1 Table — (DOCX) [file pmed.1003817.s002.docx]

| **S1a Table. Chance of reaching hypertension remission for women 2 years after surgery** | | | |
| --- | --- | --- | --- |
|  | Unadjusted OR | Adjusted OR | Adjusted –P^1^ |
| Hypertension duration > 1 year | 0.23 (0.21-0.26) | 0.39 (0.34-0.44) | <0.001* |
| Numbers of preoperative drugs |  |  |  |
| 1 | Reference | Reference | Reference |
| 2 | 0.34 (0.31-0.38) | 0.44 (0.39-0.49) | <0.001* |
| 3 | 0.19 (0.17-0.21) | 0.27 (0.23-0.31) | <0.001* |
| 4 | 0.12 (0.10–0.14) | 0.18 (0.14-0.22) | <0.001* |
| ≥5 | 0.06 (0.04-0.08) | 0.08 (0.05-0.13) | <0.001* |
| %TWL, 1 year after surgery | 1.04 (1.04-1.05) | 1.04 (1.03-1.04) | <0.001* |
| Age | 0.93 (0.93-0.94) | 0.96 (0.96-0.97) | <0.001* |
| BMI | 0.99 (0.99-1.00) | 0.98 (0.97-0.98) | <0.001* |
| Comorbid disease |  |  |  |
| Dyslipidemia | 0.46 (0.42-0.52) | 0.83 (0.74-0.94) | 0.004* |
| Depression | 0.92 (0.83-1.01) | 0.92 (0.82-1.04) | 0.186 |
| Sleep apnea | 0.76 (0.67-0.86) | 1.12 (0.97-1.30) | 0.121 |
| Diabetes | 0.60 (0.55-0.66) | 1.04 (0.92-1.17) | 0.554 |
| Cardiovascular comorbidity | 0.22 (0.17-0.29) | 0.55 (0.40-0.75) | <0.001* |
| Cerebrovascular disease | 0.42 (0.29-0.60) | 0.96 (0.61-1.50) | 0.854 |
| Education |  |  |  |
| Primary Education | 0.90 (0.83-0.99) | 1.13 (0.99-1.30) | 0.062 |
| Secondary Education | Reference | Reference | Reference |
| Higher Education | 1.00 (0.93-1.08) | 1.05 (0.94-1.17) | 0.357 |
| Surgical method |  |  |  |
| Gastric bypass | Reference | Reference | Reference |
| Sleeve gastrectomy | 0.82 (0.71-0.95) | 0.91 (0.75-1.10) | 0.316 |

1. Multivariable, logistic regression model, including all variables in the table.

- Significant value after correction with the Bonferroni-Holm method

OR = Odds Ratio (presented with 95% Confidence Interval); N = numbers; %TWL = Percentage Total Weight Loss; BMI = Body Mass Index

| **S1b Table. Chance of reaching hypertension remission for men 2 years after surgery** | | | |
| --- | --- | --- | --- |
|  | Unadjusted OR | Adjusted OR | Adjusted –P^1^ |
| Hypertension duration > 1 year | 0.20 (0.17-0.23) | 0.48 (0.40-0.59) | <0.001* |
| Numbers of preoperative drugs |  |  |  |
| 1 | Reference | Reference | Reference |
| 2 | 0.36 (0.31-0.42) | 0.49 (0.41-0.58) | <0.001* |
| 3 | 0.16 (0.13-0.19) | 0.24 (0.19-0.29) | <0.001* |
| 4 | 0.09 (0.07–0.11) | 0.13 (0.10-0.17) | <0.001* |
| ≥5 | 0.05 (0.03-0.07) | 0.09 (0.06-0.13) | <0.001* |
| %TWL, 1 year after surgery | 1.05 (1.04-1.06) | 1.05 (1.04-1.06) | <0.001* |
| Age | 0.93 (0.92-0.94) | 0.96 (0.96-0.97) | <0.001* |
| BMI | 1.01 (1.00-1.02) | 0.97 (0.96-0.98) | <0.001* |
| Comorbid disease |  |  |  |
| Dyslipidemia | 0.47 (0.41-0.53) | 0.91 (0.78-1.08) | 0.284 |
| Depression | 0.89 (0.74-1.07) | 0.96 (0.76-1.21) | 0.740 |
| Sleep apnea | 0.73 (0.65-0.83) | 0.87 (0.74-1.01) | 0.066 |
| Diabetes | 0.61 (0.54-0.68) | 0.99 (0.84-1.16) | 0.987 |
| Cardiovascular comorbidity | 0.21 (0.16-0.28) | 0.34 (0.24-0.48) | <0.001* |
| Cerebrovascular disease | 0.35 (0.23-0.55) | 1.08 (0.64-1.85) | 0.766 |
| Education |  |  |  |
| Primary Education | 0.86 (0.75-0.98) | 0.92 (0.77-1.09) | 0.327 |
| Secondary Education | Reference | Reference | Reference |
| Higher Education | 0.84 (0.72-0.99) | 0.95 (0.78-1.15) | 0.569 |
| Surgical method |  |  |  |
| Gastric bypass | Reference | Reference | Reference |
| Sleeve gastrectomy | 0.71 (0.56-0.90) | 0.77 (0.57-1.03) | 0.083 |

1. Multivariable, logistic regression model, including all variables in the table.

- Significant value after correction with the Bonferroni-Holm method

OR = Odds Ratio (presented with 95% Confidence Interval); N = numbers; %TWL = Percentage Total Weight Loss; BMI = Body Mass Index
