# Supplementary material for: Remission, relapse, and risk of major cardiovascular events after metabolic surgery in persons with hypertension: A Swedish nationwide registry-based cohort study
Source: PLoS Med. 2021 Nov 1;18(11):e1003817. doi: 10.1371/journal.pmed.1003817 (PMC8559928; doi:10.1371/journal.pmed.1003817)
Supplement: S2 Table — (DOCX) [file pmed.1003817.s003.docx]

| **S2a Table. Factors associated with risk for relapse of hypertension for women** | | | |
| --- | --- | --- | --- |
|  | Unadjusted HR | Adjusted HR | Adjusted –P^1^ |
| Hypertension duration > 1 year | 1.66 (1.47-1.88) | 1.48 (1.29-1.70) | <0.001* |
| Numbers of preoperative drugs |  |  |  |
| 1 | Reference | Reference | Reference |
| 2 | 1.57 (1.39-1.78) | 1.42 (1.25-1.62) | <0.001* |
| 3 | 2.03 (1.74-2.36) | 1.68 (1.42-1.99) | <0.001* |
| 4 | 2.50 (1.96-3.18) | 2.35 (1.82-3.03) | <0.001* |
| ≥5 | 3.40 (2.13-5.43) | 3.00 (1.76-5.11) | <0.001* |
| %TWL 1 year after surgery | 0.98 (0.97-0.99) | 0.98 (0.98-0.99) | <0.001* |
| Age | 1.02 (1.01-1.02) | 1.01 (1.00-1.01) | <0.001* |
| BMI | 0.99 (0.98-1.00) | 1.00 (0.99-1.02) | 0.059 |
| Comorbid disease |  |  |  |
| Dyslipidemia | 1.20 (1.05-1.36) | 0.99 (0.45-1.15) | 0.852 |
| Depression | 1.12 (0.98-1.28) | 1.08 (0.94-1.24) | 0.290 |
| Sleep apnea | 1.17 (0.99-1.38) | 1.09 (0.91-1.30) | 0.354 |
| Type-2 diabetes | 1.19 (1.05-1.34) | 1.05 (0.90-1.21) | 0.541 |
| Cardiovascular comorbidity | 1.50 (1.03-2.17) | 1.13 (0.72-1.77) | 0.589 |
| Cerebrovascular disease | 1.26 (0.76-2.09) | 0.98 (0.54-1.78) | 0.949 |
| Education |  |  |  |
| Primary Education | 1.11 (0.96-1.29) | 0.98 (0.94-1.28) | 0.230 |
| Secondary Education | Reference | Reference | Reference |
| Higher Education | 0.98 (0.86-1.11) | 0.94 (0.82-1.07) | 0.331 |
| Surgical method |  |  |  |
| Gastric bypass | Reference | Reference | Reference |
| Sleeve gastrectomy | 1.38 (1.05-1.81) | 1.46 (1.08-1.96) | 0.013 |

1-Multivariable Cox regression model, including all variables in the table.

*Significant value after correction with the Bonferroni-Holm method

HR = Hazard Ratio (presented with 95% Confidence Interval); N = numbers; %TWL = percentage Total Weight Loss; BMI = Body Mass Index

| **S2b Table. Factors associated with risk for relapse of hypertension for men** | | | |
| --- | --- | --- | --- |
|  | Unadjusted HR | Adjusted HR | Adjusted –P^1^ |
| Hypertension duration > 1 year | 1.75 (1.45-2.11) | 1.37 (1.10-1.71) | 0.005 |
| Numbers of preoperative drugs |  |  |  |
| 1 | Reference | Reference | Reference |
| 2 | 1.59 (1.33-1.19) | 1.33 (1.09-1.63) | 0.006 |
| 3 | 2.53 (2.06-3.12) | 2.21 (1.75-2.79) | <0.001* |
| 4 | 2.57 (1.94-3.41) | 2.36 (1.72-3.22) | <0.001* |
| ≥5 | 3.83 (2.45-5.93) | 3.25 (2.02-5.24) | <0.001* |
| %TWL 1 year after surgery | 0.98 (0.97-0.99) | 0.98 (0.97-0.99) | 0.001* |
| Age | 1.03 (1.02-1.04) | 1.02 (1.01-1.03) | <0.001* |
| BMI | 0.99 (0.98-1.01) | 1.02 (1.00-1.03) | 0.022 |
| Comorbid disease |  |  |  |
| Dyslipidemia | 1.23 (1.06-1.44) | 0.90 (0.74-1.09) | 0.275 |
| Depression | 1.02 (0.79-1.32) | 0.97 (0.73-1.29) | 0.849 |
| Sleep apnea | 1.11 (0.93-1.32) | 0.98 (0.82-1.19) | 0.867 |
| Type-2 diabetes | 1.19 (1.02-1.39) | 1.05 (0.87-1.26) | 0.610 |
| Cardiovascular comorbidity | 1.57 (1.07-2.31) | 0.98 (0.61-1.59) | 0.941 |
| Cerebrovascular disease | 1.19 (1.16-3.24) | 1.91 (1.01-3.61) | 0.046 |
| Education |  |  |  |
| Primary Education | 1.12 (0.93-1.35) | 1.05 (0.85-1.28) | 0.668 |
| Secondary Education | Reference | Reference | Reference |
| Higher Education | 1.13 (0.91-1.39) | 0.99 (0.78-1.25) | 0.935 |
| Surgical method |  |  |  |
| Gastric bypass | Reference | Reference | Reference |
| Sleeve gastrectomy | 1.75 (1.17-2.63) | 1.54 (0.97-2.46) | 0.068 |

1-Multivariable Cox regression model, including all variables in the table.

*Significant value after correction with the Bonferroni-Holm method

HR = Hazard Ratio (presented with 95% Confidence Interval); N = numbers; %TWL = percentage Total Weight Loss; BMI = Body Mass Index
