# Supplementary material for: Remission, relapse, and risk of major cardiovascular events after metabolic surgery in persons with hypertension: A Swedish nationwide registry-based cohort study
Source: PLoS Med. 2021 Nov 1;18(11):e1003817. doi: 10.1371/journal.pmed.1003817 (PMC8559928; doi:10.1371/journal.pmed.1003817)
Supplement: S3 Table — (DOCX) [file pmed.1003817.s004.docx]

| **S3 Table. Numbers of antihypertensive drugs before and 2 years after surgery** | | | |
| --- | --- | --- | --- |
| Preoperative treatment | | Postoperative treatment | |
| Number of drugs | N (%) | Numbers of drugs | N (%) |
| 1 | 5864 (36.7%) | 0 | 3649 (62.2%) |
|  |  | 1 | 1938 (33.0%) |
|  |  | 2 | 220 (3.8%) |
|  |  | 3 | 43 (0.7%) |
|  |  | 4 | 13 (0.2%) |
|  |  | ≥5 | 1 (0.0%) |
| 2 | 4524 (28.3%) | 0 | 1676 (37.0%) |
|  |  | 1 | 1281 (28.3%) |
|  |  | 2 | 1346 (29.8%) |
|  |  | 3 | 177 (3.9%) |
|  |  | 4 | 38 (0.8%) |
|  |  | ≥5 | 6 (0.1%) |
| 3 | 3149 (19.7%) | 0 | 729 (23.2%) |
|  |  | 1 | 681 (21.6%) |
|  |  | 2 | 917 (29.1%) |
|  |  | 3 | 733 (23.3%) |
|  |  | 4 | 77 (2.4%) |
|  |  | ≥5 | 12 (0.4%) |
| 4 | 1646 (10.3%) | 0 | 250 (15.2% |
|  |  | 1 | 266 (16.2%) |
|  |  | 2 | 397 (24.1%) |
|  |  | 3 | 447 (27.2%) |
|  |  | 4 | 260 (15.8%) |
|  |  | ≥5 | 26 (1.6%) |
| ≥5 | 801 (5.0%) | 0 | 74 (9.2%) |
|  |  | 1 | 73 (9.1%) |
|  |  | 2 | 155 (19.4%) |
|  |  | 3 | 202 (25.2%) |
|  |  | 4 | 194 (24.2%) |
|  |  | ≥5 | 103 (12.9%) |
