# Supplementary material for: Remission, relapse, and risk of major cardiovascular events after metabolic surgery in persons with hypertension: A Swedish nationwide registry-based cohort study
Source: PLoS Med. 2021 Nov 1;18(11):e1003817. doi: 10.1371/journal.pmed.1003817 (PMC8559928; doi:10.1371/journal.pmed.1003817)
Supplement: S4 Table — MACE, major adverse cardiovascular event. (DOCX) [file pmed.1003817.s005.docx]

| **S4 Table. Factors associated with risk for MACE** | | | |
| --- | --- | --- | --- |
|  | Unadjusted HR | Adjusted HR | Adjusted –P^1^ |
| Remission of hypertension | 0.42 (0.33-0.53) | 0.60 (0.47-0.77) | <0.001* |
| Age | 1.06 (1.05-1.07) | 1.04 (1.03-1.06) | <0.001* |
| BMI | 0.99 (0.98-1.01) | 1.00 (0.98-1.02) | 0.862 |
| Sex |  |  |  |
| Female | Reference | Reference | Reference |
| Male | 1.01 (1.67-2.49) | 1.66 (1.35-2.04) | <0.001* |
| Comorbid disease |  |  |  |
| Dyslipidemia | 2.03 (1.67-2.46) | 1.01 (0.80-1.27) | 0.953 |
| Depression | 0.89 (0.68-1.17) | 1.03 (0.78-1.36) | 0.818 |
| Sleep apnea | 1.44 (1.15-1.82) | 1.00 (0.79-1.27) | 0.980 |
| Type-2 diabetes | 2.01 (1.65-2.43) | 1.40 (1.13-1.73) | 0.002* |
| Cardiovascular comorbidity | 3.58 (2.79-4.58) | 1.93 (1.43-2.59) | <0.001* |
| Cerebrovascular disease | 3.62 (2.45-5.35) | 1.62 (1.05-2.51) | 0.030 |
| Education |  |  |  |
| Primary Education | 1.56 (1.25-1.96) | 1.31 (1.04-1.64) | 0.020 |
| Secondary Education | Reference | Reference | Reference |
| Higher Education | 0.81 (0.62-1.05) | 0.83 (0.64-1.09) | 0.189 |

1-Multivariable Cox regression model, including all variables in the table.

*Significant value after correction with the Bonferroni-Holm method

HR = Hazard Ratio (presented with 95% Confidence Interval); BMI = Body Mass Index
