# Supplementary material for: Remission, relapse, and risk of major cardiovascular events after metabolic surgery in persons with hypertension: A Swedish nationwide registry-based cohort study
Source: PLoS Med. 2021 Nov 1;18(11):e1003817. doi: 10.1371/journal.pmed.1003817 (PMC8559928; doi:10.1371/journal.pmed.1003817)
Supplement: S5 Table — (DOCX) [file pmed.1003817.s006.docx]

| **S5 Table. Factors associated with risk for mortality** | | | |
| --- | --- | --- | --- |
|  | Unadjusted HR | Adjusted HR | Adjusted –P^1^ |
| Remission of hypertension | 0.51 (0.41-0.62) | 0.71 (0.57-0.88) | 0.002* |
| Age | 1.06 (1.05-1.07) | 1.05 (1.04-1.06) | <0.001* |
| BMI | 1.00 (0.99-1.02) | 1.01 (1.00-1.03) | 0.155 |
| Sex |  |  |  |
| Female | Reference | Reference | Reference |
| Male | 2.14 (1.78-2.57) | 1.98 (1.63-2.40) | <0.001 |
| Comorbid disease |  |  |  |
| Dyslipidemia | 1.73 (1.44-2.07) | 0.99 (0.80-1.23) | 0.948 |
| Depression | 1.13 (0.89-1.43) | 1.34 (1.05-1.71) | 0.018 |
| Sleep apnea | 1.35 (1.08-1.69) | 0.93 (0.74-1.18) | 0.561 |
| Type-2 diabetes | 1.76 (1.46-2.11) | 1.30 (1.06-1.60) | 0.011 |
| Cardiovascular comorbidity | 2.24 (1.70-2.94) | 1.27 (0.93-1.74) | 0.134 |
| Cerebrovascular disease | 2.60 (1.71-3.95) | 1.62 (1.02-2.56) | 0.039 |
| Education |  |  |  |
| Primary Education | 1.16 (0.92-1.45) | 0.95 (0.76-1.20) | 0.693 |
| Secondary Education | Reference | Reference | Reference |
| Higher Education | 0.88 (0.69-1.11) | 0.90 (0.71-1.14) | 0.389 |

1-Multivariable Cox regression model, including all variables in the table.

*Significant value after correction with the Bonferroni-Holm method

HR = Hazard Ratio (presented with 95% Confidence Interval); BMI = Body Mass Index
