# Supplementary material for: Remission, relapse, and risk of major cardiovascular events after metabolic surgery in persons with hypertension: A Swedish nationwide registry-based cohort study
Source: PLoS Med. 2021 Nov 1;18(11):e1003817. doi: 10.1371/journal.pmed.1003817 (PMC8559928; doi:10.1371/journal.pmed.1003817)
Supplement: Attachment — Submitted filename: stenberg.pdf [file pmed.1003817.s008.pdf]

This paper uses routine datasets to assess the linkage between metabolic surgery and subsequent hypertension and other events.

I was asked for a statistical report and I interpret that to include all aspects of the design and conduct of the study.

Points of detail

Page 8 Strictly speaking Figure 1 is part of the results so I would call it out there and not in the methods.

Page 10 Is EBMIL actually used later? I am not suggesting it should be, in fact there are three variables here which must be interrelated, but if it was planned to be analysed and is now not presented we should perhaps be told why.

Page 10 I think it would be better throughout to use a notation other than $\pm$ as it is used ambiguously for standard deviations, standard error, confidence interval.

Page 13, Supplementary Figure 1 The issue here is change over time so I would have presented this as a table cross-classified by number before and number after. Then we could see how many moved from each category to each. It would also show how many were taking more after than before. This would help people who would like to analyse specific changes and also subsequent meta–analysts who could extract what ever they want.

Supplementary Tables There is no limit as far as I know on the number of tables so since these seem to report important secondary outcomes why not move them into the main body of the article? This is a genuine question, I do not have strong feelings either way. These tables do not use the same covariates as the other tables. Perhaps explain in the methods why this is?

Point of more substance

In Table 2 we see that the unadjusted and adjusted odds ratios for sex were quite different (0.82 versus 1.20). This can happen of course but the implication is that the other variables in the model may be unbalanced between the sexes and possibly that the drivers of the effect may also differ. I wonder therefore whether it would be enlightening to split the sample by sex and

check whether the same variables appear in both sexes. The results in Table 3 are less startling but checking there might be worth doing.

Summary

Mostly minor points for clarification.

Michael Dewey
