# Supplementary material for: Remission, relapse, and risk of major cardiovascular events after metabolic surgery in persons with hypertension: A Swedish nationwide registry-based cohort study
Source: PLoS Med. 2021 Nov 1;18(11):e1003817. doi: 10.1371/journal.pmed.1003817 (PMC8559928; doi:10.1371/journal.pmed.1003817)
Supplement: Attachment — Submitted filename: finalresponse_plos.docx [file pmed.1003817.s009.docx]

Dear Editor,

Thank you for your positive response, and for giving us the opportunity to resubmit our manuscript. We have to the best of our ability addressed the final requests and comments point-by-point below and accordingly made changes throughout the manuscript.

All authors have contributed to this revision and approved the final version of the manuscript.

Please do not hesitate to contact us if further modifications are needed.

On behalf of all authors - sincerely,

Erik Stenberg MD, PhD

Requests from Editors:

Thank you for your attention to our initial requests. Please respond to the following before we proceed:

1) There are some instances of the term "subject." In Fig 1 title, please replace "subject" with participant, patient, individual, or person

*Authors response: All remaining “subjects” terms are now removed (except for those referring to another study where the term was used; i.e. “The Swedish Obese Subjects study (SOS-study)”.*

2) In the discussion, please replace “compliance” with adherence

*Authors response: Compliance has now been replaced with adherence as requested.*

3) Please include line numbers in your next draft

*Authors response: Line numbers have been included in the revised manuscript*

Comments from Reviewers:

Reviewer #1: The authors have addressed all my points.

In response to an editorial request the authors have now adjusted for multiplicity. It is not clear to me what the logic behind this is nor exactly how it has been done. Has the adjustment been applied across all the analyses in the paper? That seems clearly wrong. Is it applied on the family of comparisons in a single table so in Table 2 the family size is 18 if I count them correctly. In that case why would you want to adjust each for all of them? Or has it been done separately for the set of comparisons per covariate. so for comorbid disease the family size is 6?

I think that at the very least the procedure needs to be clarified but more importantly it needs to be justified scientifically or clinically, and if not removed.

*Authors reply: The adjustments for multiple calculations were introduced in response to a previous request. The adjustments were made separately for each table. In the present form the unadjusted p-value is presented with “*” symbolizing significance after adjustment. In our opinion the adjustment is motivated by the high numbers of evaluated factors. In response to this comment, we have further clarified the analyses in the methods section*

Reviewer #2: Dear authors,

Thanks for your detailed responses.

I have a few suggestions to improve your manuscript:

1.	Table 1: line: Hypertension duration > 1 year, n (%) 0 (0.0%) 13268 (83.0%) 4406 (70.1%) 8862 (91.4%)

I think the percentages of remissions columns should refer to the 13268 subjects, like the entire table.

*Authors response: This is a good point that makes the results of Table 1 more easily interpreted. In response to this comment, the percentages for remission and non-remission has been updated to refer to the numbers of patients with each condition at each line. (changes to Table 1)*

2.	You found that males have more chance to reach remission. Can you speculate the reason because it occurs? Central obesity? Do you have data from abdominal circumference?

*Authors response: While we agree that this is an interesting point to learn more of, the study was not designed to specifically evaluate the cause of sex-related differences. We believe that this should be a focus of future research and would therefore prefer not to speculate further on the cause of these differences.*

3.	BMI is associated with a lower remission rate. It would be interesting if you could show a cutoff value. Is it possible?

*Authors response: We agree that this is an interesting point. However, this was not part of our study design and should also rather be the focus of future research.*

Reviewer #3: Thank you for your thoughtful and thorough reply and appropriate revision in response to my initial review. This reviewer has no further comments or suggestions.
